# The Anatomy and Physiology of the Liver

**Published:** 1834-07-01

**Authors:** 


*The Anatomy and Physiology of the Liver.* By FRANCIS KIERNAN, Esq., M.R.C.S. [From the *Philosophical Transactions.*]—London, 1833. 4to. pp. 60.

THE minute anatomy of morbid structures has of late years been prosecuted with great assiduity. While we do not agree with those who think such researches frivolous, we may be forgiven for doubting whether they will ever be productive of the practical advantages expected from them. It is much to be lamented that an equal share of attention has not been bestowed on the minute anatomy of healthy organs, from which, in our opinion, much more important results are likely to follow, since it has rarely happened that a laborious investigation of the intimate structure of any organ has not conduced, in some degree, to the elucidation of its functions. All that is most certain in physiology has been derived from this source; nor, in the conduct of such inquiries, should any peculiarity of structure, however apparently insignificant, be considered unworthy of attention. A careless anatomist, for example, in examining the valves of the veins, might content himself with looking at a few of them, and, finding them all to be merely reduplications of the internal membrane, and all nearly alike, might think their further investigation superfluous. Harvey was at the trouble of tracing them throughout, and found indeed that they were all alike in a most important particular—they all leaned the same way; and it was mainly on this fact that he built one of the most splendid discoveries ever made in science.

We have derived much pleasure and instruction from the perusal of Mr. Kiernan's paper. His anatomy of the liver is by far the most complete and satisfactory hitherto published; and, in giving to the world the results of his own observation, he has been careful to ascertain what had been done by his predecessors: a species of diligence which cannot be too much commended, and in which it were to be wished that he had more companions; the maxim "decies repetita placebit" having been rather too extensively adopted by the medical discoverers of the present day.

The substance of the liver, as is well known, consists of a congeries of small lobules; and the most interesting part of the paper before us refers to the distribution of the blood-vessels and ducts in and around these lobules: of this we shall endeavour to give a brief abstract, afterwards quoting some passages in illustration of other points in the anatomy and physiology of the organ. We may premise that, if, in so intricate an anatomical description, we should, in any instance, misconceive our author's meaning, we are, saving our infallibility, open to correction.

The vessels of the liver are—

I. The HEPATIC VEINS, which consist of

1. The *intra-lobular* hepatic veins, which are contained within the lobules: one of these occupies the centre of each lobule, and receives the blood from four or six smaller branches which terminate in it. The intra-lobular veins correspond in their ramifications with the form of the lobules whose substance is placed around them; and, as will presently be shown, they receive the blood from a plexus formed in the lobule by the *portal vein.*

2. Hepatic veins, which are contained in *canals* formed by the lobules: these, for the sake of perspicuity, we shall call

*canalicular* veins. The formation of the canals differs according to the relation of the contained vessel to the intra-lobular veins: where the intra-lobular terminate directly in the canalicular vein, the canal of the latter is necessarily formed by the bases of the lobules resting upon it, and the contained vein is termed *sublobular;* where the canalicular vein is not formed immediately by the intra-lobular, but by the junction of several other canalicular veins, the canal containing it is formed by a tubular inflection of the surface of the liver; so that, in the one case the canals are formed by the continuity of the *bases* of the lobules, and in the other, by the continuity of those surfaces which will be presently described as *capsular*.

II. The PORTAL VEINS, HEPATIC DUCTS, and HEPATIC ARTERIES, which must be described together, because they accompany each other throughout their course, being all contained in the *portal canals.* These canals begin at the transverse fissure, where they are continuous with the concave surface of the liver; and, like those of the larger hepatic veins, they are formed by the *capsular* surfaces of a certain number of the lobules.

To understand the distribution of the vessels contained in these canals, we must trace the course of *Glisson's capsule*. The liver is invested by a membrane, which stands related to it much as the pia mater does to the brain: it is a cellulo-vascular membrane, which is reflected inwards at the transverse fissure, and encloses in a sheath the portal veins, the hepatic arteries, the ducts, the nerves, and the absorbents: it is here that it is called the capsule of Glisson, in the ordinary language of anatomists. A continuation of this membranous sheath accompanies the contained vessels to their minutest ramifications; it enters the interlobular fissures, and, with the vessels, forms the *capsules* of the lobules; it finally enters the lobules, and, with the blood-vessels, expands itself over the secreting biliary ducts. Hence arises a natural division of the capsule into three portions—a *vaginal,* an *inter-lobular,* and a *lobular* portion.

At the transverse fissure, the duct, vein, and artery, divide into branches which enter the *portal canals,* invested with the above-described membrane, the membrane lining the canals and enclosing the vessels. These branches, again, divide and subdivide into smaller branches, which enter smaller canals, and every canal, however small, contains one principal branch of each of these vessels; frequently, however, two ducts and two arteries are contained in the same canal.

To the larger vessels the terms *hepatic ducts, portal veins,* and *hepatic arteries,* may be restricted, in order to distinguish them from the branches. The excreting ducts are composed of the hepatic ducts contained in the canals, of their vaginal branches, also contained in canals, and of the interlobular branches, which, arising from the vaginal branches, ramify in the interlobular fissures. The interlobular ducts enter the lobules, in which they form plexuses: these plexuses may be called the *lobular-biliary,* or secreting biliary plexuses, the ducts composing them being the secreting organs of the bile. The excreting ducts and their branches are invariably accompanied by the arteries and portal veins, and their branches, the former conveying blood to their coats, the latter conveying it from them. A duct is never unaccompanied by an artery and a vein, the vein being always a branch of the portal. The veins and arteries also enter the lobules: the veins form plexuses, the branches of which terminate in the intra-lobular hepatic veins; and from the blood circulating through these plexuses the bile is secreted. The lobular arteries are exceedingly minute, and few in number: they are the nutrient vessels of the lobules, and probably terminate in the plexuses formed by the portal vein. From the ducts, veins, and arteries, therefore, three sets of branches arise, namely, the *vaginal,* the *interlobular,* and the *lobular* branches.

Such is the best account we are able to give of the distribution of the vessels of the liver, according to Mr. Kiernan. We fear that the want of engravings may prevent our description from being as perspicuous as we could wish; but we shall not attempt to illustrate the matter further, since, as Hudibras sagely observes,

> " brevity is very good,
> When w' are, or are not, understood."

Want of space obliges us to omit many of the able anatomical descriptions with which Mr. Kiernan's paper abounds: we subjoin, however, a few extracts, chiefly with the view of inducing the reader to peruse the entire memoir, which will not fail to reward him amply for his trouble.

In the following passage our author explains the manner in which the veins of the liver inosculate with each other.

" Ramifying in fissures which are continuous with each other throughout the whole liver, the interlobular branches of the portal vein anastomose freely with each other, enveloping every lobule in a venous web: the intralobular branches of the hepatic veins, on the contrary, confined within the lobules, have no direct communication with the corresponding branches of the surrounding lobules, from which they are separated by the substance of these bodies, and by the intervening interlobular ducts, veins and arteries, situated in the interlobular fissures; and one intralobular vein can be injected from another only through the medium of the intervening portal veins. When injected with mercury or size, the intralobular veins appear in the centres of the lobules in the form of points, stellæ, or twigs; these veins, therefore, unlike the interlobular portal veins, do not anastomose with each other. If, by means of a pipe and glass tube, mercury be thrown into a large hepatic vein on the surface of a section, it will return by several smaller neighbouring hepatic veins, which, generally, are branches descending to terminate in the larger vessel in which the pipe is fixed. If these vessels be tied, the mercury will return by large hepatic veins, situated at a distance from the injected vessel, and not branches of it. In this experiment the force used is not sufficient to propel the mercury through the intervening intralobular, lobular and interlobular veins, and by such means to form a communication between the two hepatic veins; for if force sufficient to effect this were used, the mercury would return by portal as well as by hepatic veins, which is not the case. If the inferior cava be opened at its posterior part, and if by the same means mercury be thrown into a small hepatic vein, it will immediately return to the cava by other hepatic veins, without appearing in the superficial intralobular veins, and without passing into portal veins. From these experiments, it appears that the sublobular veins anastomose with each other, and that their intralobular branches do not.

"The most superficial vessel, the intralobular branches of which are seen on the surface, should be chosen for these experiments, and, in order to ascertain that the mercury does not pass into the portal vein, a section of the liver should be made at some distance below the injected vessel. A glass tube and pipe, whereby the pressure may be graduated, should be always used in these experiments. Size injected into one hepatic vein will always, and wax will sometimes, return by other hepatic veins.

"By contrasting the hepatic veins with the portal vein, we find that no two intralobular branches of the former anastomose with each other; that the interlobular branches of the latter form one continuous plexus throughout the whole liver; that the sublobular veins anastomose directly, and not through the medium of the intralobular branches; that the portal veins have no direct communication with each other, but anastomose by means of their interlobular branches; that the hepatic veins, like the other veins of the body, proceed in a direct course to their termination in the cava; that the portal vein, accompanied by an artery, resembles an artery in its ramifications; that the larger hepatic veins, having longitudinal fibres in their coats, differ in structure from the portal vein; and that the blood contained in the liver after death is almost invariably found in the hepatic veins, the portal vein being usually empty." (P. 736.)

From the double venous circulation carried on within the liver, it results that this organ is naturally in a state of sanguineous congestion. "Hence," says our author,

"arises the great difficulty of making successful injections of the human liver: the plexuses may, however, be frequently well, but seldom equally, injected, and always with greater success from the portal, than from the hepatic vein, the latter, and those portions of the plexuses immediately surrounding its intralobular branches, generally containing whatever blood may remain in the liver after death. The plexuses may be always injected with facility from the portal or hepatic veins, and the injection will pass freely from one vein into the other without extravasation, if the liver has been previously deprived of all its blood by the ligature of the portal vein and hepatic artery in the living animal. Anatomists have considered, that the free communications which exist between the two vessels obviate the difficulty which would otherwise arise in the circulation through the liver, from the want of power consequent on the presence of the two veins; and, although the communications between these vessels appear, upon experiment, to be more free than those which exist between the hepatic artery and the portal vein, and between arteries and veins generally in other parts of the body, yet it appears that the arteries and veins in the spleen and kidney, and probably in all glands, communicate with equal freedom. The lobular venous plexus is best examined in the superficial lobules; but here again the human liver presents a difficulty, particularly in the adult, in consequence of the opacity of the proper capsule: the liver of the cat, and that of the smaller animals generally, appear to have no cellular capsule, and are consequently more favorable for this purpose.

"The venous plexus ramifies on the biliary plexus: the blood circulating through it is composed of the portal blood, and certainly of that portion of the arterial blood which, having nourished the excreting ducts and supplied them with mucus, and having circulated through the vasa vasorum of all the vessels, becomes venous, and is received into the branches of the portal vein, by which, with the portal blood, it is conveyed to the plexus; and from this mixed blood the bile is secreted." (P. 745 )

Anatomists have long described two distinct substances in the liver, one red, and the other yellow: Mr. Kiernan, however, denies the validity of this distinction, affirming that the substances are essentially the same, and that the red colour is produced merely by congestion. After a full detail of the opinions of preceding anatomists, he continues,

"My attention was first directed to the anatomy of the liver by the study of the admirable works of M. Andral. In the first organs I examined, I found the small branches of the hepatic veins ramifying exclusively in the red, and those of the portal vein in the yellow substance. I concluded that the liver was composed of two venous trees, a portal and an hepatic tree, the former having a cortex of yellow, the latter of red substance; and with M. Boulland I thought it probable that the red substance was the organ of the function imagined by Bichat. I next ascertained the lobular structure, and concluded with Ferrein that the red substance was medullary, and the yellow cortical. Subsequent dissections, in which I found branches of both the portal and hepatic veins ramifying in the red substance, tended to unsettle the opinions I had formed respecting the anatomy and physiology of the two substances; and these opinions were finally overturned by the examination of a liver in which I found the branches of the portal vein alone ramifying in the red, and those of the hepatic veins in the yellow substance. The only conclusion that could be drawn was, that the red colour resulted from congestion; that it was medullary, occupying the centre of each lobule, when the hepatic, and cortical, forming the circumference, when the portal vein was congested. It occurred to me that the kidneys of birds having, like the liver, a double venous circulation, were equally subject to congestion, and would, like it, present an appearance of two substances. Dissection verified this conjecture; but the apparently two substances are red, one, however, being of a much deeper colour than the other. I have satisfied myself by repeated injections, by examination with the microscope, and by experiments on living animals, that the lobules are of the same structure throughout; that one portion of a lobule is not more vascular than another; that the acini of Malpighi, by contrast with the congested vessels, are even more apparent in the red than in the yellow substance; and that these supposed two substances are consequently identical in structure. That secreting biliary ducts are contained in the red as well as in the yellow substance, is proved by the relation given by M. Andral of a case of jaundice with 'coloration insolite du foie.' 'Foie volumineux, pesant, très-dur, se déchirant difficilement, offrant une teinte générale d'un brun verdâtre. En l'examinant avec plus d'attention, on trouve que cette teinte n'est pas uniforme, et que le parenchyme du foie est formé, 1°. par un tissu d'un blanc verdâtre, disposé sous forme de lignes ou de plaques irregulières (c'est le tissu blanc ordinaire hypertrophié); 2°. par un tissu d'un vert brun foncé, duquel dépend la coleur générale que présente le foie, et qui est l'analogue du tissu rouge ordinaire.' This was a case of vitiated biliary secretion, with general biliary and partial sanguineous congestion. The ordinarily yellow substance was of a greenish white colour, being congested with greenish bile only; the ordinarily red substance was of a deep brownish green, this colour evidently resulting from biliary and sanguineous congestion combined. I have met with more than one case of this kind; I have also seen cases of jaundice in which there was no biliary congestion of the liver, and the highest state of biliary congestion without jaundice. In attempting to estimate the causes of the various shades of colour observed in the liver, it is not sufficient to examine the cystic bile alone; the hepatic bile should be also examined; and it will be generally found, as in the above case, that these shades of colour depend either on biliary or sanguineous congestion alone, or on the various combinations of both." (P. 752.)

The following are our author's conclusions with respect to the function exercised by the liver.

"The physiological deductions arising out of the preceding anatomical facts are extremely simple. If it could be shown that two substances exist in the liver, it might be fairly presumed that this organ executes two functions; but each lobule being, in itself, a perfect gland, and of the same structure throughout, each lobule, and consequently the whole liver, executes but one function, the secretion of bile.

"It has been shown that all the vasa vasorum of the liver are branches of the hepatic artery and portal vein; that branches of the portal vein arise in the coats of the hepatic veins themselves; and that the veins of the coats of the vessels constitute the hepatic origin of the portal vein. The arterial blood having circulated through the coats of the vessels, becomes venous, and is conveyed by the veins arising in the coats of the vessels into those branches of the portal vein which correspond to the vessels in the coats of which the veins, arise: thus, from the coats of the vaginal ducts, veins, and arteries, they convey the blood into the vaginal veins; and from the coats of the interlobular ducts, veins and arteries, into the interlobular veins. From the coats of the hepatic veins and inferior cava, the blood is conveyed into the interlobular portal veins. In the vaginal and interlobular veins, the blood conveyed from the coats of the vessels becomes mingled with the proper portal blood. This mixed blood is conveyed by the interlobular veins into the lobular venous plexuses, in which the lobular arteries probably terminat[illegible] after having nourished the secreting ducts. From the mixed [illegible] circulating through the plexuses, the bile is secreted by the lobular or secreting biliary plexuses.

"The blood which enters the liver by the hepatic artery fulfils three functions: it nourishes the liver; it supplies the excreting ducts with mucus; and, having performed these purposes, it becomes venous, enters the branches of the portal vein, and contributes to the secretion of the bile. The portal vein fulfils two functions; it conveys the blood from the artery, and the mixed blood to the coats of the excreting ducts. It has been called the vena arteriosa, because it ramifies like an artery, and conveys blood for secretion; but it is an arterial vein in another sense, being a vein to the hepatic artery, and an artery to the hepatic vein. The hepatic veins convey the blood from the lobular venous plexuses into the vena cava inferior." (P. 754.)

The paper concludes with some remarks on the opinion of those physiologists who hold that the bile is secreted by the hepatic artery, and an account of an interesting dissection which the author had an opportunity of making, of a child in which Mr. Abernethy found the portal vein terminating in the inferior cava. On this head, however, we must refer the reader to the paper itself; in concluding our notice of which, we have only to regret that our own limits, and the nature of the subject, have not allowed us to do justice to its merits. It is a learned, concise, and well-digested memoir, replete with original observation, and is in all respects highly creditable to Mr. Kiernan's abilities both as an anatomist and a writer.

The work is illustrated with some neat engravings, from drawings by the author, by the aid of which the anatomical details, though necessarily minute and intricate, are for the most part rendered perfectly intelligible.

